# Automated Personalized Goal Setting for Individual Exercise Behavior: Protocol for a Web-Based Adaptive Intervention Trial

**DOI:** 10.2196/73766

**Published:** 2025-11-12

**Authors:** Juan Carlos Caro, Phuong H Nguyen, Stefan Lipman

**Affiliations:** 1 Department of Industrial Engineering Faculty of Engineering University of Concepción Concepcion, null Chile; 2 School of Health Policy and Management Erasmus University Rotterdam Rotterdam The Netherlands

**Keywords:** contextual bandits, behavior change, adaptive experiments, personalized exercise, machine learning, user autonomy

## Abstract

**Background:**

The incidence of chronic diseases associated with physical inactivity is on the rise, being one of the leading risk-increasing factors for early death rates throughout the world. Often, physical activity interventions fail to deliver sustained adherence over time due to limiting tailoring to individual baseline characteristics, leaving out contextual changes over time. One solution for this issue may be the use of adaptive interventions relying on contextual multiarmed bandits, a type of reinforcement learning algorithm, that can use baseline and contextual individual data to personalize aspects of the intervention, such as developing personalized workout plans.

**Objective:**

The main objectives of this study are (1) to determine the effectiveness of contextual bandits for automated goal setting in the context of a web-based physical activity intervention, (2) to understand the role of user characteristics impacting ideal workout schedules based on adherence to predetermined goals, and (3) to explore the influence of user autonomy on recommendation effectiveness.

**Methods:**

We developed a protocol for a web-based adaptive intervention trial to investigate the effectiveness of goal recommendation (task difficulty) based on reinforcement learning. The web application (named Apptivate) creates workout routines with 3 different difficulty levels, changing the total workout duration as well as rest times between exercises. Physical activity professionals validated the routine design, ensuring that workouts match recommended guidelines for healthy adults. An initial pilot was conducted, aiming for 800 university students to test the web application for 1 week, to provide initial data to calibrate the algorithm as well as overall feedback for the web application. For the main study, a total of 500 university students will be recruited to participate for 40 days during early 2026. Participants will be divided into 3 groups: user choice (no recommendation), user choice with automated recommendations (contextual bandits), and automated plans without choice.

**Results:**

The pilot was conducted in September 2025. Data analysis for the pilot is undergoing, and the main study is planned for early 2026. Our main statistical analysis includes a direct comparison (paired tests) between success rates across intervention arms, as well as by difficulty level and individual characteristics.

**Conclusions:**

Physical activity maintenance is key to achieving long term health goals. Tailored digital interventions are promising strategies for physical activity adherence, but personalization often fails to consider dynamic contextual changes. The proposed protocol for a physical activity intervention using adaptive experimentation can provide robust causal inference on the role of choice versus autonomy when goal difficulty is tailored under an adaptive data-driven approach.

**International Registered Report Identifier (IRRID):**

PRR1-10.2196/73766

## Introduction

Regular physical activity (PA) is essential for maintaining overall health and well-being [1]. Extensive research has demonstrated that consistent exercise is associated with numerous health benefits, including improved sleep quality, enhanced mood, and a reduced risk of noncommunicable chronic diseases such as diabetes, stroke, depression, and dementia. In contrast, physical inactivity has been identified as one of the leading risk factors for premature mortality and a significant contributor to the rising health care costs [2-4]. Despite the well-documented benefits of PA and risks associated with a sedentary lifestyle, 31% of the global adult population—equivalent to 1.8 billion adults—fail to meet the recommended levels of PA [5]. Even with unwavering global efforts from national and international policy, mass media campaigns, and social support and educational programs [6], the number of adults who are physically inactive has been steadily increasing in high-income countries [7].

To address this public health issue, researchers have designed and implemented various interventions aimed at promoting regular exercise adherence, many of which are based on prominent social and psychological theories [8,9], such as theory of planned behavior [10] or self-determination theory [11]. Although these interventions have shown promise in increasing individuals’ intentions to engage in PA, a key challenge lies in translating these intentions into sustained behavior [12,13]. This discrepancy is known as the “intention-behavior gap,” emphasizing that goal intentions only partially materialize on corresponding actions [14]. Studies indicate that individual factors such as lack of motivation and low self-efficacy, as well as environmental barriers, such as time constraints, can hinder individuals from acting on their intentions, even when they recognize the benefits of PA [13,15]. Moreover, these factors are highly heterogeneous across individuals and time. Thus, addressing these challenges requires individualized, flexible, and engaging interventions that consider individual needs, preferences, and contexts, and that promote self-efficacy and focus on closing the intention-behavior gap.

Mobile apps have emerged as promising platforms for delivering effective health interventions owing to widespread technological access and potential for personalization or tailoring [16]. While the overall evidence on the effectiveness of app-based mobile health (mHealth) interventions on PA engagement is mixed [17-19], interventions that allow for tailoring (ie, fitting interventions to users), especially considering changes in context over time, show promising results [16]. For example, a study in 2006 demonstrated that computer-tailored interventions, which provide personalized feedback and actionable recommendations, significantly increased PA levels among participants compared to generic advice [20]. Similarly, researchers in 2011 found that interventions incorporating goal-setting, self-monitoring, and personalized planning were more effective in promoting sustained behavior change [21]. These approaches recognize that a “one-size-fits-all” strategy is often insufficient, as individuals face unique challenges such as time constraints, lack of social support, or low self-efficacy. By addressing these barriers through tailored solutions, such as adaptive goal-setting, context-specific reminders, or individualized support systems, interventions can better align with participants’ lifestyles and may potentially enhance their ability to act on their intentions [22,23].

**Figure 1:**
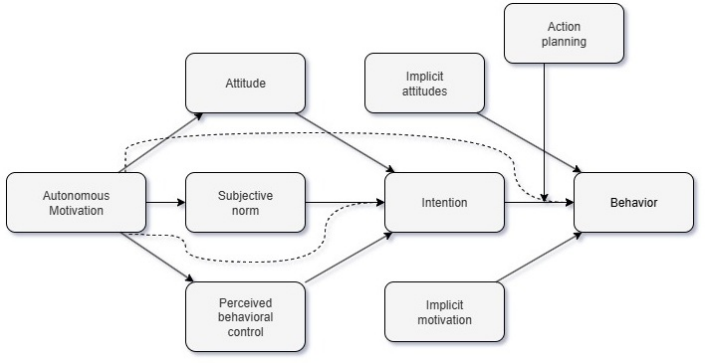
Integrated behavioral change model (source: Hagger and Chatzisarantis [26]).

The taxonomy of personalization features is extensive, as apps allow for data-driven mechanisms that can respond to user behavior and real-time context, delivering messages, adapting to user experience, tailoring interactions with other participants, and providing user targeting, among other features [23]. However, in most apps, personalization is designed to increase user engagement with the app rather than focusing on sustained behavior adoption [24], which may be a consequence of lacking integration between app-based health research and existing theoretical frameworks explaining behavior adoption [25]. Evidence from such behavioral models, such as the integrated behavior change (IBC) model [26,27], propose that behavior adoption requires sustained effort, that is, a sustained focus on realizing behavior change goals (ie, plans) over time (Figure 1). Therefore, one way to potentially enhance the effectiveness of tailoring in app-based interventions is to focus on such goals, with personalized goals that are selected to fit individuals’ unique profile (eg, adapting difficulty to individuals’ preferences or characteristics) in order to maximize the likelihood of goal completion over time. Such personalization should acknowledge time-variant factors (or contexts) that can affect individuals on a daily basis, as well as time-invariant aspects that could vary dramatically across users. Earlier work on message tailoring has suggested that including elements of multiple dominant psychological theories into tailoring approaches increases overall effectiveness [28], suggesting the IBC model, which integrates across the major dominant psychological theories [26,27], is well-suited for developing tailoring approaches.

With technological advances in artificial intelligence, personalization has become easier to implement in dynamic settings, where users are interacting repeatedly with tailored components. In particular, contextual multiarmed bandits, or simply contextual bandits (CBs), have become an effective reinforcement (machine) learning personalization tool in the health behavior domain, where treatment (option) recommendations are optimally determined to maximize user performance using individual characteristics and behavior [29]. In short, CB algorithms use all available data to predict the action that would provide the best outcome to a given user, while learning from the relative performance of all possible actions. In experimental literature, there is a novel approach using CBs named adaptive interventions, emphasizing the idea that we can pose a sequential field experiment with an adaptive sampling procedure that aims to determine which types of individuals benefit from certain intervention arms over time, while also learning about the average effectiveness of each arm [30,31]. Adaptive interventions allow us to estimate the relative effectiveness of each arm while providing the optimal path for each user, based on their expected behavioral response. Overall, CBs have important advantages as an automated personalization tool, requiring less data to determine which interventions are more effective overall and allowing an understanding of which characteristics are most likely to influence individuals’ performance.

Adaptive interventions using CB algorithms have become popular in the public health domain, with promising results [16,23,32]. Systematic reviews for adaptive interventions targeting PA and sedentary behavior reveal that most studies focus on tailored feedback as the main personalization component, with few interventions focusing on goal tailoring. Moreover, studies designed with goal setting features mostly focus on difficulty progression over time (eg, increasing duration or reducing resting time) or alternating types of exercises (eg, cardio, strength, and flexibility) based on the user’s health profile [23]. In particular, while difficulty progression seems reasonable as mastery increases, evidence on motivation shows that dynamic environmental factors can strongly influence goal completion as difficulty increases [33]. Altogether, to date, it is still unclear to what extent automated personalized goal difficulty recommendations over time and individual preferences influence health behavior adoption.

While machine learning approaches such as CBs offer promise by matching individuals based on their characteristics or context, this optimal recommendation could be seen as a directed action (data-driven), or a recommendation, mediated through individual choice. This is particularly relevant in the context of exercise, where preferences for workout intensity and capabilities to do more demanding exercises can differ widely between individuals [34]. Enabling respondents to choose their workouts, rather than be assigned one through machine learning, respects these diverse preferences and could enhance intervention effectiveness. That is, according to self-determination theory, an influential psychological theory of motivation, being able to choose enhances autonomy, a key driver of intrinsic motivation [35]. Furthermore, evidence suggests that individuals may benefit from a process of self-experimentation to find what works best for them [36]. A meta-analysis shows the potential of choice-based interventions in the context of health behaviours (including exercise), showing improved adherence and reduced dropout across various health behaviors [37]. Past research has found that providing individuals with choice in PA positively influences their perception of exercise [38,39], general well-being [40], as well as adherence to the intervention regime [41]. However, enabling choice is not without risks; individuals may opt for interventions that are ineffective for them due to, for instance, misjudging their own self-control [42] or selecting appealing but ultimately ineffective strategies [43]. Thus, in the context of machine learning–based tailoring, incorporating a “choice” arm allows for the investigation of how algorithmically assigned interventions compare with those selected by individuals themselves, enabling a direct comparison between data-driven assignment of exercise goals versus self-chosen goals.

This study explores three research questions to evaluate the effectiveness of adaptive interventions that focus on goal difficulty and user choice:

1. Can CBs outperform performance success rate (goal completion) relative to user-chosen workouts?
2. Which user characteristics are most relevant for determining choice probabilities among intervention arms?
3. To what extent does user autonomy in choosing a workout plan influence the effectiveness of recommendations based on CBs?

By addressing these questions, this experiment aims to contribute to the growing body of research on adaptive behavior change interventions. Understanding the interplay between user choice and data-driven recommendations can inform the development of more effective mHealth apps [44].

## Methods

### Apptivate

For this project, the experiment will be conducted using the web application “Apptivate” [45], extending its initial functionally to incorporate CB algorithms connected to a routine difficulty assignment. Apptivate creates workout routines with 3 different difficulty levels, changing the total workout duration as well as rest times between exercises. PA professionals validated the routine design, ensuring that workouts match recommended guidelines for healthy adults. The overall design allows users to conduct exercises without equipment following the in-app instructions, covering different muscle groups as well as providing both warm-up and stretching exercises.

Apptivate adopts the IBC model for PA [26] as a framework for tailoring workout difficulty recommendations (ie, goals). By leveraging the IBC model, the app ensures that its recommendations address not only dynamic changes in behavior over time but also the psychological factors that influence behavior change. In particular, we capture data on the psychological constructs defined in the IBC model, which are later used as contexts for the algorithm learning process. Participants that take part in the main study will be assigned to 1 of 3 conditions:

- Choice-driven: Users select their desired workout difficulty level from a predefined menu. This approach provides users with a sense of autonomy and control over their exercise routine. Choice-driven tailoring leaves the selection of the intervention to the user, allowing individuals to reveal their preferences in a participatory process [33].
- Data-recommended: The CB algorithm determines optimal workout difficulty (based on user data and the IBC model) and provides a recommendation to the user, but the user retains the option to choose an alternative. This design allows us to investigate the interplay between user autonomy and the effectiveness of data-driven recommendations [27].
- Data-driven: The CB algorithm automatically recommends workouts based on user data, including contextual features based on the IBC model, without any opportunity to opt into a different difficulty level. This method leverages a machine learning approach to personalize workout plans and potentially optimize user performance (measured as goal completion).

**Figure 2:**
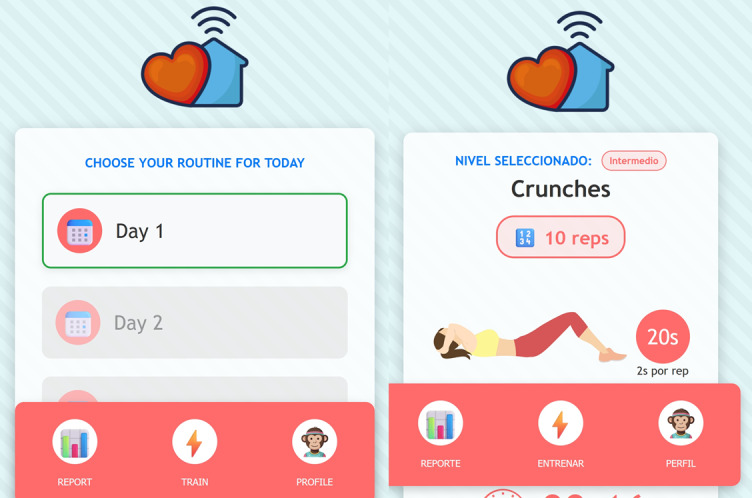
Apptivate main menu and workout view.

Apptivate also includes several personalization features to enhance user engagement [15,23]. Users can change their username and avatar, as well as opt for timed notifications. The system includes motivational messages, a progress tracker, and routine feedback (Figure 2).

### Contextual Bandits Algorithm

**Figure 3:**
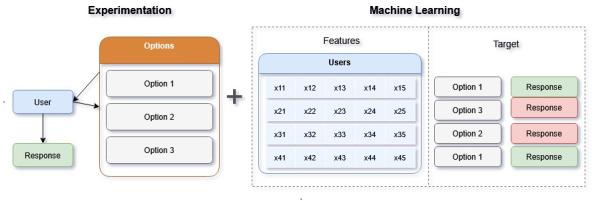
Contextual bandit algorithm design.

The contextual multiarmed bandit model used in this study is a type of reinforcement machine learning approach, which uses experimentation to learn about users’ performance and recommend the best potential option. As shown in Figure 3, the algorithm incorporates users’ past outcomes (target) and context (features) to assign probabilities to each treatment option and recommend the best possible treatment in the future (higher likelihood of success, noted in green). Once given a recommendation, users respond (either positively or negatively in this case), and this information is used for further training the algorithm for the next interaction. There are several algorithms developed to solve CB models, differing in how probabilities for each option are updated (learning) and the degree of exploration (randomness) involved in providing users with different options.

Formally, at each round *t*=1*,...,T*, we observe the individual’s context (*x_t_* ∈ X), from which the algorithm recommends an action (*a_t_* ∈ A), and the environment reveals a reward (*r_t_* ∈ [0*,*1]). Intuitively, the algorithm needs to choose the policy *π* ∈ Π that maximizes the cumulative reward , where Π is a mapping of all policies relating context to actions (Π ⊆ {X → A}). We can define the average reward of a policy *π* as:

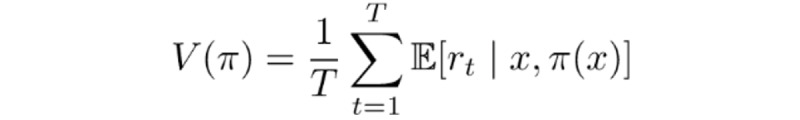



Based on this idea of expected cumulative reward, we can then determine the cumulative regret from a given policy as the difference between the observed reward for the recommended action and the expected reward of the recommended action:

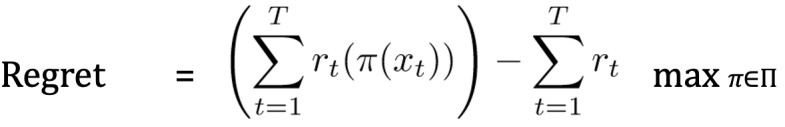



In this setup, the different types of CB algorithms differ in their proposed approaches to achieve regret minimization. In practice, some algorithms will be better suited for different empirical applications, depending on the number of rounds, the possible set of actions, and the heterogeneity in the context from which the algorithm learns to allocate the best recommendation [31]. As such, the pilot phase is critical to determining the best approach for this experiment.

### Study Design

This study poses two main hypotheses: (1) goal personalization with CBs decreases the intention-behavior gap for some users, and (2) the effectiveness of such goal personalization on behavior is mediated through individual choice. To explore these hypotheses, participants will be randomly assigned to 1 of 3 groups: choice-driven, data-recommended, or data-driven.

The target population are university students (both undergraduate and graduate) from different institutions, allowing for differences in region, language, and time zone of the participants. Randomization will be stratified by baseline demographic factors, including household composition, income, age, and gender, to ensure a representative sample within each institution. The inclusion criteria require participants to be physically healthy adults (aged older than 18 years), with access to a smartphone compatible with the mobile web application. Exclusion criteria include significant physical disabilities or medical conditions limiting exercise, based on the Physical Activity Readiness Questionnaire [46], as well as individuals actively participating in competitive sports. The participant assignment procedure will use block randomization to reduce bias and ensure even group distribution, with allocations concealed and blinded from both participants and researchers through a secure system managed by the data specialist team. Data will be stored at a secure server at the University of Concepcion, with backups of the database each hour. A deidentified version of the data and dictionary can be made available upon request.

Since there is no prior evidence regarding effect size for this study, sample size was determined using 1000 simulations, following existing guidelines [47]. Sample sizes were estimated considering the particularities of adaptive experiment design. Simulations based on the relevant populations were conducted using the epsilon-greedy algorithm. Based on simulated data, a sample size of 500 participants during 10 rounds is sufficient to evaluate differences between intervention arms and conduct statistical inference, particularly regarding demographic and behavioral characteristics between participants. The authors are currently working on a publication describing the simulations and associated power analysis, which will become available in late 2025.

To train the reinforcement learning algorithm for the study, initial data are required for which a pilot stage was designed. In this stage, 800 participants will be recruited at one university in order to provide 1 week of activity using the app (two 3-day rounds with a rest day in between). In order to determine the sample size for the pilot, standard power calculation was assumed with a uniform distribution over 3 arms (standard randomized controlled trial). With 665 observations, the pilot is sufficient to detect differences between users. The data from round 1 will be used to estimate the optimal arm for round 2, which will be used for calibration. Participants complete a comprehensive baseline survey eliciting the IBC model as well as several other relevant characteristics (eg, time and risk preferences; see Multimedia Appendix 1) and then are assigned randomly between the 3 difficulty levels for the first round. After completion, participants are compensated for their time. Individuals can opt out from the study at any time.

During the main part of the study, 500 participants will be recruited from multiple sites expecting to participate in a medium-term challenge, completing 10 rounds, equivalent to 39 consecutive days using the app (including rest days). As noted earlier, this provides 5000 unique examples for the algorithm to determine optimal policy over time. Users will be allocated to each arm based on their baseline survey and the trained algorithm during the pilot stage. Regardless of their initial difficulty assignment, each participant will be randomly allocated with equal probability between (1) choice-driven, (2) data-recommended, and (3) data-driven arms. Participants will be compensated for their time, and there will be additional prizes using a lottery mechanism that favors (in probability) those users completing more consecutive workout routines over the course of the study. In both the pilot and main experiment, participants will stay engaged via direct email, push notifications, and social media campaigns.

### Data Collection

Data collection in this study spans multiple dimensions, capturing individual characteristics, app usage patterns, and most importantly, user performance. This multifaceted approach allows us to provide insights on the potential heterogeneous impact of tailored goal-setting interventions.

- Baseline survey: Participants will complete an online survey to gather demographic information (eg, age, gender, and socioeconomic status), fitness history (eg, frequency and type of PA), and constructs from the IBC model, such as perceived behavioral control, motivation types (autonomous and controlled), and self-regulatory capacities.
- Fitness app data: The app will track participant engagement and outcomes, including goal completion rates, total time spent on physical activities, and engagement metrics (eg, frequency of app interactions).
- Algorithm data: The CB algorithm embedded in the app will collect data on contextual features (eg, user preferences, past performance, and fitness level) and reward functions to predict optimal goals and adapt recommendations over time.

### Statistical Analysis

Statistical analysis will focus on comparing goal completion rates, engagement metrics, and user satisfaction across the 3 intervention groups. To address missing data, a multiple imputation approach will be applied to ensure robustness in outcome estimates. The primary analysis will estimate the conditional average treatment effect for each intervention arm, with subgroup analyses to identify variations by demographics, baseline fitness, and psychological factors. Mixed-effects models will account for repeated measures and clustering within participants, while sensitivity analyses will assess the robustness of the findings under varying assumptions.

During the pilot phase, multiple CB algorithms will be evaluated based on metrics such as regret minimization (difference between actual and optimal rewards) and balance between exploration and exploitation. The main experiment will focus on estimating the conditional average treatment effect and analyzing differences in goal completion rates and engagement metrics. Subgroup analyses will explore how demographic characteristics (eg, age and gender), motivational factors, and baseline fitness levels influence responsiveness. Metrics such as cumulative reward, prediction accuracy, and contextual feature importance will be analyzed to evaluate the algorithm’s ability to personalize recommendations effectively.

### Ethical Considerations

Our study is considered an adaptive experiment or adaptive intervention trial [30,31]. Unlike a randomized controlled trial, sampling is not fixed, but instead each round is controlled by a recommendation algorithm, as noted in the previous section. As such, based on institutional guidelines, this study does not meet the criteria for a clinical trial. The Ethics, Bioethics, and Biosafety Committee of the Vice Presidency of Research and Development at the University of Concepción has reviewed our study under Project Fondecyt (11240325), titled “Tailored goals versus individual choice: a field experiment on commitment devices for health behavior,” submitted in the Fondecyt Initiation 2024 Call by Dr Juan Carlos Caro Seguel, as the lead researcher and faculty member at the School of Engineering at the University of Concepción. The Committee has confirmed that it meets the ethical and bioethical standards and procedures established nationally and internationally for research involving human participation. Although this protocol does not correspond to a clinical trial, we included a SPIRIT (Standard Protocol Items: Recommendations for Interventional Trials) checklist to facilitate study implementation (Multimedia Appendix 2).

## Results

This research is supported by the Fondecyt Initiation Grant 2024 (grant ANID 11240325; funding period 2024-2026). The pilot was conducted in September 2025. Data analysis for the pilot is undergoing, and the main study is planned for early 2026. Preliminary results from the pilot data will be available in November 2025. Our main statistical analysis includes a direct comparison (paired tests) between success rates across intervention arms, as well as by difficulty level and individual characteristics. We anticipate publishing the findings in late 2026. Dissemination will be conducted simultaneously via a web-based seminar for all participant institutions during 2026.

## Discussion

### Anticipated Findings

This study introduces a novel approach to promoting sustained PA through a mobile app, leveraging CB learning for personalized goal difficulty recommendations, under an IBC framework. By combining this theory-informed machine learning approach with different levels of user autonomy, the intervention addresses critical gaps in existing mHealth literature, particularly the role of autonomy and motivation on personalized goal setting. We expect to find heterogeneity in both (1) individual behavioral responses to personalized goals, and (2) autonomy preferences for choice versus automation. Such heterogeneity should align with the IBC model and reflect the particular hurdles of motivation versus goal difficulty.

Findings from this field experiment will contribute to the growing field of adaptive digital health technologies, offering practical insights for scaling effective interventions for sustained health behavior. Ultimately, this research has the potential to inform public health strategies by providing evidence on the interplay between user autonomy and data-driven recommendations, fostering more engaging and effective health behavior change digital tools.

There are limitations to this study. First, participant dropout may introduce bias if attrition is nonrandom, reducing overall statistical power. Our sample size considers a maximum 20% dropout rate, to maintain statistical validity. Second, findings may not generalize to populations of different ages or health statuses or to those without smartphone access and from other cultural contexts beyond those included in the study. In an effort to provide better external validity, we aim to implement this study in countries with different languages and cultural backgrounds.

### Conclusions

PA maintenance is key to achieving long term health goals. Tailored digital interventions are promising strategies for PA adherence, but personalization often fails to consider dynamic contextual changes. The proposed protocol for a PA intervention using adaptive experimentation can provide robust causal inference on the role of choice versus autonomy when tailoring goal difficulty is done under an adaptive data-driven approach. Dissemination plans will focus both on participants and the scientific community, and all participatory events are designed to be conducted simultaneously via a web-based seminar for all participant institutions during 2026.

## Appendix

Multimedia Appendix 1 Baseline questionnaire.

Multimedia Appendix 2 SPIRIT 2025 checklist of items to address in a randomized trial protocol.

Multimedia Appendix 3 Peer review report by INTERTRANSDISCIPLINARY Evaluation Group, FONDECYT, Agencia Nacional de Investigación y Desarrollo, (ANID; National Agency for Research and Development, Chile).

## Abbreviations

CB: contextual bandit
IBC: Integrated Behavior Change
mHealth: mobile health
PA: physical activity
SPIRIT: Standard Protocol Items: Recommendations for Interventional Trials
