# Supplementary material for: Automated Personalized Goal Setting for Individual Exercise Behavior: Protocol for a Web-Based Adaptive Intervention Trial
Source: JMIR Res Protoc. 2025 Nov 12;14:e73766. doi: 10.2196/73766 (PMC12658392; doi:10.2196/73766)
Supplement: Multimedia Appendix 1 [file resprot_v14i1e73766_app1.docx]

Multimedia Appendix 1: Baseline questionnaire.

| QuestionID | Question_EN | AnswerValues_EN |
| --- | --- | --- |
| parQ_1 | Have you ever been diagnosed with a heart condition by a doctor, who recommended that you only engage in physical activity supervised by healthcare personnel? | Yes;No |
| parQ_2 | Do you experience chest pains caused by physical activity? | Yes;No |
| parQ_3 | Have you noticed chest pain caused by physical activity? | Yes;No |
| parQ_4 | Do you tend to faint, or lose balance, as a result of dizziness? | Yes;No |
| parQ_5 | Has a doctor ever prescribed any medication for high blood pressure or other cardiovascular problems? | Yes;No |
| parQ_6 | Do you have any bone or joint disorders that could worsen due to physical activity? | Yes;No |
| parQ_7 | Do you have knowledge, either through personal experience or advice from a doctor, of any other physical reason that would prevent you from exercising without medical supervision? | Yes;No |
| athlete | Do you participate in any sports at a professional level? | Yes;No |
| age | What is your age? | 18 - 100 |
| language | Language | ES;EN;NL |
| name | Name |  |
| age | What is your age? | 18 - 100 |
| gender | How would you describe yourself? | Male; Female; Non-binary; Other |
| employment | What best describes your employment status over the last three months? | Working part-time; Working sporadically; Not working; Other |
| marital_status | What is your current marital status? | Married; Cohabiting; Widowed; Divorced/Separated; Never been married |
| household_1 | Including yourself, how many people live in your household? | 1 - 10 |
| household_2 | How many children under the age of 18 live in your household? | 1 - 10 |
| income | What is your monthly income range? | 0 - 3.000.000 or more (intervals of 300.000) |
| current_exercise | How many days per week do you engage in moderate to vigorous physical exercise (e.g., jogging, swimming, cycling, or gym workouts) | 1 to 7 |
| ibc_controlled_motivation | **Please indicate below whether or not the statements below are true for you** |  |
| ibc_cm1 | I work out because other people say I should. | 1-5: Not at all true - Very true |
| ibc_cm3 | I work out because others will be disappointed if I don’t. | 1-5: Not at all true - Very true |
| ibc_cm8 | I feel under pressure from my friends/peers/partner to work out. | 1-5: Not at all true - Very true |
| ibc_autonomous_motivation | **Please indicate below whether or not the statements below are true for you** |  |
| ibc_am_1 | It is pleasurable to work out. | 1-5: Not at all true - Very true |
| ibc_am_2 | It is important to me to work out. | 1-5: Not at all true - Very true |
| ibc_am_3 | I enjoy working out | 1-5: Not at all true - Very true |
| ibc_attitude_header | **For me, working out during the next four weeks would be…** |  |
| ibc_attitude_1 | Unimportant - Important | 1 to 7 |
| ibc_attitude_2 | Not worthwhile - Worthwhile | 1 to 7 |
| ibc_attitude_3 | Harmful - Beneficial | 1 to 7 |
| ibc_subjective_norm | **Do you agree with the following statements?** |  |
| ibc_sn_1 | Most people who are important to me would want me to work out during the next four weeks. | 1-7: Disagree very strongly - Agree very strongly |
| ibc_sn_2 | Most people I know would approve of me working out during the next four weeks. | 1-7: Disagree very strongly - Agree very strongly |
| ibc_sn_4 | Most people who are relevant to me would approve of me working out during the next four weeks | 1-7: Disagree very strongly - Agree very strongly |
| ibc_perceived_behavioural_control | **Do you agree with the following statements?** |  |
| ibc_pbc_2 | It is mostly up to me whether or not I work out during the next four weeks | 1-7: Disagree very strongly - Agree very strongly |
| ibc_pbc_3 | If I wanted to, I could work out during the next four weeks. | 1-7: Disagree very strongly - Agree very strongly |
| ibc_pbc_4 | Working out during the next four weeks is up to me. | 1-7: Disagree very strongly - Agree very strongly |
| ibc_intention | **Do you agree with the following statements?** |  |
| ibc_intention_1 | I intend to work out during the next four weeks. | 1-7: Disagree very strongly - Agree very strongly |
| ibc_intention_2 | I plan to work out during the next four weeks. | 1-7: Disagree very strongly - Agree very strongly |
| ibc_intention_3 | I will try to work out during the next four weeks. | 1-7: Disagree very strongly - Agree very strongly |
| time_preference_1 | I am generally an impatient person | 1-7: Disagree very strongly - Agree very strongly |
| time_preference_2 | I am generally an impulsive person | 1-7: Disagree very strongly - Agree very strongly |
| risk_preference | I am generally a person who is fully prepared to take risks | 1-7: Disagree very strongly - Agree very strongly |
| need_for_autonomy_1 | If I had to change my behaviour to get healthier, I would motivate myself | 1-7: Disagree very strongly - Agree very strongly |
| need_for_autonomy_2 | If I had to change my behaviour to get healthier, I would ask family and friends to motivate me | 1-7: Disagree very strongly - Agree very strongly |
| need_for_autonomy_3 | If I had to change my behaviour to get healthier, I would ask an expert to motivate me | 1-7: Disagree very strongly - Agree very strongly |
