# Supplementary material for: Automated Personalized Goal Setting for Individual Exercise Behavior: Protocol for a Web-Based Adaptive Intervention Trial
Source: JMIR Res Protoc. 2025 Nov 12;14:e73766. doi: 10.2196/73766 (PMC12658392; doi:10.2196/73766)
Supplement: Multimedia Appendix 3 [file resprot_v14i1e73766_app3.pdf]

Translated from Spanish to English - www.onlinedoctranslator.com

Agencia Nacional de Investigación y Desarrollo
Ministerio de Ciencia, Tecnología, Conocimiento e Innovación

ANID/Projects Subdirectorate/No. 80

Santiago, February 1, 2024
Ref.: Project No. 11240325

Dear Mr. JUAN CARO:

On behalf of the National Agency for Research and Development, I am writing to inform you that your project No. 11240325, submitted to the 2024 Fondecyt Research Initiation Competition in the INTER-TRANSDISCIPLINARY Evaluation Group, has been approved.

In this call, 1,400 projects were submitted, of which 420 (30.0%) were awarded.

In the INTER-TRANSDISCIPLINARY Group, 72 proposals were submitted, and 23 were awarded (31.9%). Their project received 5th place and a score of 4.175.

Following this letter, you will find the ratings and comments your project received, along with a certificate certifying its award. You will also find the following information in the Online Evaluation System:

a. Approved budget for each year of execution, with the possibility of redistributing the funds allocated annually, if deemed appropriate.
b. Authorizations-Certifications Report, if applicable. Indicate the documents that must be submitted to initiate the project, within a period of three months from the date of this letter (section 11.2.1. literal d) of the competition rules).
c. "Decision to Execute" button. When pressed, you must indicate whether you accept or reject the project award within a maximum of 10 administrative business days from the date of this letter.

Regarding the process of Signing the Agreement and Transferring Resources, please review the instructions available at InstructionsSignatureConvenioIniciacion2024.pdf . and please note that any queries or requests related to the execution of your project should be sent through ANID Help (https://ayuda.anid.cl ).

Please accept our congratulations in advance for this important achievement in your scientific career.

Best regards,

Camilo Erazo Leiva
Deputy Director(s)
Subdirectorate of Research Projects
National Agency for Research and Development

**RESULT OF THE EVALUATION PROCESS**

**RATINGS OBTAINED BY PROJECT NO. 11240325**

| Stage 1: Quality, Feasibility and Scientific or Technological Novelty of the Proposal (75%) | Stage 2: Academic and Research Career of the Applicant (25%) |
|---|---|
| 4.0 | 4.7 |

**EVALUATION COMMENTS**
**PROJECT NO. 11240325**

Note:

The comments issued by the Evaluation Group may highlight aspects that it deems necessary to convey to the applicants, in accordance with section 9.6 of the competition rules.

## 1. QUALITY, FEASIBILITY AND SCIENTIFIC OR TECHNOLOGICAL NOVELTY OF THE PROPOSAL

Contextualization of the proposal
Obesity is on the rise worldwide, particularly due to the combination of a sedentary lifestyle and a nutrient-poor diet. It is also associated with non-communicable diseases, detrimental mental health, and contributes to the global burden of disease. In Chile, more than two-thirds of the adult population is overweight or obese, and fewer than one in ten people achieve the minimum required levels of physical activity.

Despite policy efforts targeting obesogenic environments, individual behavior change is critical to achieving long-term population health goals. A promising area of research into interventions that can promote behavior change is the work done on commitment devices, which allow individuals to voluntarily commit to future actions, with a specific consequence (negative or positive) linked to success or failure.

Worldwide, the prevalence of insufficient physical activity has increased dramatically in recent decades, particularly in high-income countries. Widespread sedentary lifestyles combined with low-nutritional diets have contributed to the sustained rise in obesity worldwide, accelerating in recent years due to the COVID-19 pandemic. An important question is what drives an individual to adhere to an exercise routine. While some people function well under expert advice, others require autonomy. With the advancement of artificial intelligence, dynamic recommendations are possible, particularly using the machine learning tool called "contextual bandits (CB)," which delivers recommendations that maximize users' performance given their individual characteristics and behavior.

Adaptation in physical activity interventions has been widely used, particularly in mHealth interventions. Research suggests that effective interventions using engagement devices (such as preset goals) should incorporate adaptation—that is, personalizing goals and/or incentives based on user behavior and characteristics. With technological advances in artificial intelligence, personalization has become easier to implement in dynamic environments, where users repeatedly interact with tailored components (e.g., goals, messages) during the intervention. In particular, contextual bandits (CBs) have emerged as an effective machine learning personalization tool in the health behavior field, where goal recommendations are optimally determined to maximize user performance using individual characteristics and behavior, with the potential to eliminate choice altogether (i.e., automated goal assignment).

There are several knowledge gaps regarding the role of automated goal-setting recommendations for physical activity. This project proposes a field experiment to test three empirical questions regarding automated goals as commitment devices for promoting health behavior change:

The working hypotheses are:
1. Users who receive automated goal recommendations based on individual behavior (goal competency) and characteristics (context) will have a higher goal completion rate relative to users with random recommendations.
2. Some users will benefit from choice when offered a personalized recommendation, while others will perform better with automated goals without choice.
3. Default user characteristics (context) will predict which types of users are more likely to achieve positive outcomes in each arm.

Goals
The overall objective is to study the role of automated personalized goals and individual choice in physical activity behavior using a contextual bandit algorithm.

The specific objectives are:1. To determine the contextual bandit algorithm that provides the best offline prediction of goal success in each treatment arm.2. To compare the effectiveness of automated recommendations over random choice toward individual goal completion across multiple rounds.3. To observe differences in goal performance between users presented with automated recommendations and participants presented with automated goals without choice.4. To explore the contextual determinants of effectiveness in each treatment arm.
Design:
Once participants are recruited, they are given an online baseline questionnaire to collect their demographic characteristics and measurements. This is a randomized clinical trial with three treatment arms: randomized recommendations, automated recommendations, and automated targeting.
1. Random recommendations. (data-driven recommendation). Choice-driven approach.

2. Automated recommendations - CB optimal recommendations - Data-driven approach
3. automated objectives - CB automated (without choice). Data-driven approach

Automated arms 2 and 3 are implemented using the adaptive experimental framework, where sampling in each round is based on outcomes and context using CB. In both phases, participants are presented with three possible fitness challenges (goals) varying in difficulty (easy, medium, and hard). Once a goal is selected, they must perform the corresponding exercise routine on a fitness app for three consecutive days (with one day of rest between each round).

The study is divided into two phases: a pilot study and the main experiment. In both phases, participants are presented with a menu of three possible fitness challenges (goals) that vary in difficulty (easy, medium, and hard). Once a goal is chosen, participants must perform the corresponding exercise routine on a fitness app for three consecutive days (with one day of rest between each round). Once participants are recruited, they are presented with an online baseline questionnaire to collect their demographic characteristics, as well as measures of several key aspects that we expect to be correlated with goal competition—i.e., contexts.
Phase 1: 4-day pilot, 1,000 participants
Phase 2: Adaptive experiment 40 days 500 participants

Based on previous studies, they will use a professional sample collection company (e.g., Lucid) to ensure a balanced and representative participant pool. The researchers receive anonymized data from a third-party recruitment company, Qualtrics, and the fitness app, and the algorithm connects directly to both platforms via Python, allowing the CB algorithm to operate autonomously.

Inclusion criteria: Only the ability to provide informed consent and a basic understanding of how to use the fitness app, as well as a mobile device to install and operate the software.
Participants receive a reward for the time spent completing the questionnaires (in addition to the lottery incentive at the end of the second phase).

Expected results:
Comparing these two approaches is particularly pertinent because of their diametrically opposed ethical and practical implications. In data-driven adaptation, responsibility for the final assignment can be removed entirely from the user or presented as a recommendation, preserving autonomy. In choice-based adaptation, responsibility rests entirely with the individual, based on their own judgment of the target's performance.
Choice-based personalization does not require information, but data suggest that people's preferences for interventions may not match their interests.
Second, they aim to provide novel evidence on which contextual factors determine user performance.
They could contribute to optimizing goal-setting mechanisms in physical activity apps. This has substantial implications for practitioners, policymakers, and users. The general application of the knowledge gained from this experiment could substantially increase the adherence and performance of individuals who plan to engage in physical activity behaviors.
Second, while the experiment focuses on a specific behavior (physical activity), our design allows us to understand in its entirety which individual factors make some users more likely to assume optimal recommendations based on their own data. These tests can have substantial implications in multiple contexts where policymakers seek to push

people toward specific behaviors by setting optimal (or optimally recommended) goals.

This is a well-written project that addresses the problem of physical activity adherence from an innovative and interdisciplinary perspective. It presents a comprehensive conceptual framework, providing readers with a review of the state of the art on the topic of behavior change related to physical activity.

Furthermore, the state of the art recognizes the heterogeneity in individual responses and indicates the knowledge gaps that exist. These gaps motivate the working hypotheses, which in turn imply the following specific objectives:
1. Determine the contextual bandit algorithm that provides the best prediction of target success in each treatment arm.
2. Compare the effectiveness of automated recommendations over random selection toward achieving individual goals over multiple rounds.
3. Observe the differences in goal achievement performance between users who receive automated recommendations and participants who receive automated goals without choice.

4. Explore the contextual factors that determine efficacy in each treatment group.

This panel considers one of the weaknesses of this proposal to be the very limited specification of the fitness app. The RFP indicates that a software developer will be employed only for the first year (will it be perfect in the first year and never need modifications?). And it doesn't indicate what features are expected of this app (will it work for Apple and Android?). The "Resources Available" section states that the sponsoring institution has researchers who can assist in content creation, but the application itself doesn't mention anything about the content itself. Furthermore, while a company can help recruit as homogeneously as possible, there's always the potential for participation bias (what's valid for our volunteer might not be valid for the rest of the population). Another weakness is that the justification for the requested resources doesn't specify the type of students being targeted (undergraduate vs. graduate) and what program they're studying.

We also recommend that the applicant better explain the available resources (especially in a project like this, which requires both content creation and technical feasibility). The submitted document is very repetitive.

The theoretical and conceptual foundations and state of the art seem too extensive, but it is coherent, up-to-date and well presented.

General objective: Its general objective is clear and coherent and the specific objectives are appropriate.

Methodology:
• Although the objectives seem appropriate, as does the method of testing them through a clinical trial. However, it does not comply with the basic methodology for developing a clinical trial nor does it avoid potential biases.
• Does not mention the ethical aspects of developing a randomized trial
• It does not refer to the randomization mechanism, concealment of the sequence, or masking of participants or outcome assessors.

• Does not explain how it will handle losses, dropouts, or loss of follow-up in any of the trial branches.
• It is not explained how the participants' consent will be applied.
• A prize is offered at the end of the essay, which could be difficult for the ethics committee evaluating the project. It is suggested that you explore how to approach this.

Data analysis: Not described. Infrastructure and resources requested are adequate.

Scientific or technological novelty of the proposal: The proposal is novel, but it should be carried out more in accordance with universally accepted parameters for conducting clinical trials. It is multidisciplinary. It is very well written.

**2. ACADEMIC AND RESEARCH CAREER OF THE APPLICANT**

The IR defines himself as a social scientist with fifteen years of research experience in complex data analysis and behavioral modeling. Since 2022, he has been an assistant professor at the Faculty of Engineering, University of Concepción. He currently resides in Chapel Hill, United States. He holds two degrees: a degree in commercial engineering in 2008 and a degree in culinary arts in 2012, the latter obtained in Philadelphia, United States. He also holds a master's degree in economics (University of Chile, 2012) and a doctorate in health policy and administration (University of North Carolina, 2020). He also mentions postdoctoral research with the University of Luxembourg, which allowed him to delve deeper into core areas of the field. He led an ANID project on COVID-19 entitled "Artificial Intelligence to Promote Self-Efficacy among Primary Caregivers of Children" and is co-PI on a Stanford University project close to application. He teaches and has taught well-documented courses related to his topic. Good V and very aligned with your proposal.
In his application, he only lists three published articles, despite having many more. The information the candidate uploads to the platform is evaluated.

In Contribution or Connection with Society and the Environment, he indicates that he received an Oxfam scholarship to contribute to the dialogue on gender segregation in the workplace. He also presented a presentation on the tax on sugary drinks to the Senate. He also volunteered for UN fundraising and provided technical assistance for a school meal program during his doctorate. He successfully integrates his training as a chef into networks linked to child health, a topic on which he focuses his doctoral research. He also highlights his extensive dedication to providing technical assistance to public institutions, especially the Ministry of Education in the area of school meals.

Agencia
Nacional de
Investigación
y Desarrollo

Ministerio de Ciencia,
Tecnología, Conocimiento
e Innovación

**AWARD CERTIFICATE**
**FONDECYT RESEARCH INITIATION COMPETITION**

February 1, 2024

Camilo Erazo Leiva, Deputy Director (s), Research Projects Subdirectorate, of the National Agency for Research and Development, certifies that Mr. JUAN CARLOS CARO SEGUEL, has awarded project N°11240325 in the FONDECYT Research Initiation Project Competition 2024, entitled TAILORED GOALS AND INDIVIDUAL CHOICE: A FIELD EXPERIMENT ON AUTOMATED COMMITMENT DEVICES FOR HEALTH BEHAVIOR.

The project, sponsored by the UNIVERSITY OF CONCEPCION, has a duration of 3 years – from March 15, 2024 to March 14, 2027 –, and funding of $34,800,000, $34,200,000 and $32,160,000, for each year of execution, respectively.

This certificate is issued to the interested party for the purposes he deems appropriate.

Camilo Erazo Leiva
Deputy Director(s)
Subdirectorate of Research Projects
National Agency for Research and Development

Moneda nº1375,
Santiago, Chile
Tel: 56 (2) 2365 4600
www.anid.cl
@AnidInforma



Gobierno de Chile
